# Precocious puberty and Chiari I malformation with syrinx: a case report of an unusual presentation of Costello syndrome

**DOI:** 10.1186/s13633-019-0067-8

**Published:** 2019-10-22

**Authors:** Naomi S. Schwartz, Molly O. Regelmann

**Affiliations:** 1 Department of Pediatrics, Children’s Hospital at Montefiore, Albert Einstein College of Medicine, Bronx, NY USA; 2 Division of Pediatric Endocrinology & Diabetes, Children’s Hospital at Montefiore, Albert Einstein College of Medicine, 3415 Bainbridge Ave, Bronx, NY 10467 USA

**Keywords:** Costello syndrome, Precocious puberty, Chiari I malformation, Syrinx

## Abstract

**Background:**

Costello syndrome (CS) is a rare RASopathy causing developmental delays, short stature and classically, delayed puberty. We present a patient with CS and central precocious puberty (CPP).

**Case presentation:**

A female patient with CS presented at 6 years 10 months of age with breast development. CPP was biochemically confirmed at 7 years 1 month of age, no additional pituitary dysfunction was noted and puberty progressed at follow-up. Brain magnetic resonance imaging (MRI) revealed a Chiari I malformation with a syrinx, requiring surgical decompression. The patient was successfully treated with histrelin.

**Conclusions:**

Although recent publications do not recommend routine brain MRI in girls with isolated CPP over 6 years of age, in those with CS actionable MRI findings are more likely and imaging should be performed. It is unclear whether the cerebral malformation in the patient contributed to CPP or was an incidental syndromic finding.

## Background

Costello syndrome (CS) is an autosomal dominant RASopathy with an estimated prevalence of 1:300,000 to 1:1,230,000 [1]. It is caused by a mutation in *HRAS*, a proto-oncogene encoding p21RAS proteins in the rat sarcoma – mitogen-activated protein kinase (RAS-MAPK) and phosphoinositide-3-kinase – protein kinase B (PI3K-AKT) pathways. The classic CS phenotype includes distinct coarse facial features, macrocephaly, loose skin, hypermobile joints and papillomata [1, 2]. Associated medical concerns include intellectual disability, heart defects including hypertrophic cardiomyopathy, neurological abnormalities and increased risk of malignancy [2, 3]. CS children typically exhibit growth failure in weight and length during infancy, resulting in short stature [2, 4]. Growth charts developed for CS children 0–10 years show that the 95th percentile for children with CS is consistent with the 5th percentile on the 2000 Center for Disease Control/ National Center for Health Statistics height chart, just under a 4 standard deviation (SD) difference. While there are no large rigorous studies assessing the growth hormone - insulin-like growth factor 1 (GH-IGF-1) axis in children with CS, it has been estimated GH deficiency may be present in up to 40% of these children [4].

Most patients with CS present with delayed puberty [2, 5]. In a 2005 report of adults with CS, six out of eight women displayed delayed puberty, with pubertal onset ranging from 11 to 16 years (unspecified whether onset was measured from thelarche or adrenarche). Menarche ranged from 13 to 17 years, with two patients experiencing primary amenorrhea. The report summarized prior literature of five additional CS women, three of whom either had delayed puberty or amenorrhea [5]. There have only been three prior documented cases of CS patients with precocious puberty: two males and one patient documented incidentally without gender detail [6, 7]. We present a female patient with an atypical presentation of CS associated with central precocious puberty (CPP) and a Chiari I malformation with a syrinx. The patient’s presentation emphasizes the need for pubertal assessment at a young age and the importance of neuroimaging in patients with CS.

## Case presentation

A 6 year 10 month old female of Dominican descent with known CS presented to pediatric endocrinology for consultation regarding concerns for precocious puberty. She was diagnosed with CS based on phenotypic features, and confirmatory testing at 1 year of age revealed a p.Gly12Ser missense mutation in the *HRAS* gene. Medical history included global developmental delay, gastroesophageal reflux and feeding difficulties beginning in infancy. On physical exam, height was − 3.67 SD below the mean and body mass index (BMI) Z-score was 0.65. She had Marshall and Tanner stage [hereafter referred to as Tanner stage (TS)] II breasts, TS I pubic hair, no axillary hair, and no clitoromegaly. A bone age x-ray was interpreted as 1 year advanced of her chronological age. Laboratory tests were collected in the mid-afternoon and were notable for borderline pubertal luteinizing hormone (LH) at 0.22 μIU/mL and prepubertal estradiol of 3 pg/mL; thyroid function tests and GH markers were interpreted as normal. Additional mid-afternoon testing 3 months later confirmed a pubertal LH (0.44 μIU/mL), and she demonstrated an above average height velocity of 6.2 cm/year. Ultrasensitive LH and estradiol assays were performed at the Quest Diagnostics Nichols Institute, San Juan Capristano, CA. An ultrasensitive LH value of ≥0.3 μIU/mL was interpreted to be consistent with central puberty.

There were significant challenges arranging follow-up appointments and testing. The patient next presented at 8 years 0 months with documented pubertal LH (0.62 μIU/mL), and bone age was interpreted as 8 years 10 months. Height measured − 3.17 SD below the mean and height velocity was 8 cm/year (above the 97th percentile for age). At 9 years 7 months, height measured − 2.56 SD below the mean. The bone age had advanced to 12 years, and she appeared to have progressed in puberty based on TS III breasts. Mid-afternoon laboratory tests confirmed progression of CPP with LH elevation of 2.65 μIU/mL (estradiol was 7 pg/mL; pubertal estradiol at Quest Nichols Institute is considered > 16 pg/mL. The clinical interpretation was the patient was in central puberty based on the LH and clinical signs of endogenous estrogen production, including accelerated height velocity, progression of breast development and advanced bone age); IGF-1 and insulin-like growth factor binding protein-3 were within normal limits for age, and thyroid function tests were normal.

**Fig. 1:**
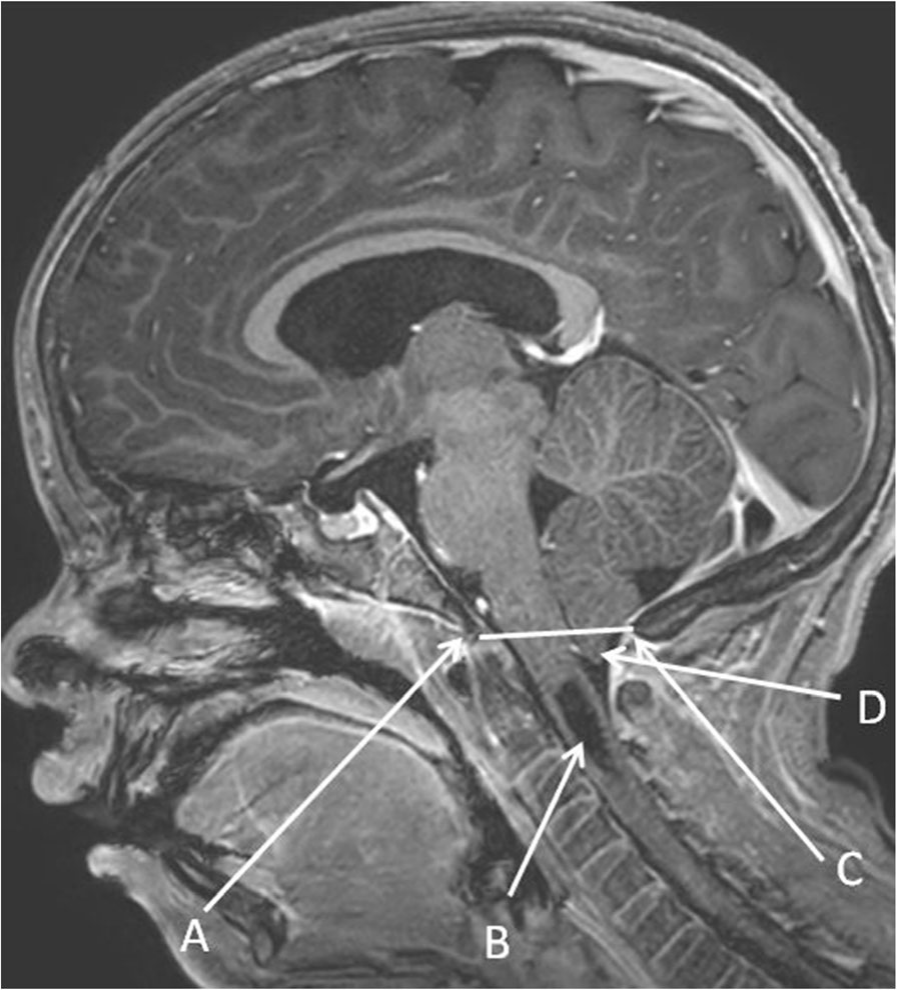
Brain MRI at 9 years 10 months, structures as labeled. Depicted is a Chiari I malformation with a syrinx at C1-C4. **a** Basion, **b** Syrinx, **c** Opisthion, **d** Low lying cerebellar tonsil

Brain magnetic resonance imaging (MRI) was performed at 9 years 10 months to investigate the patient’s CPP, as well as to screen for syndromic intracranial abnormalities. A Chiari I malformation was discovered, with cerebellar tonsils extending 7 mm below the foramen magnum. There was also an associated 8 mm anterior-posterior × 8 mm transverse × 3 cm craniocaudal syrinx at the level of C1-C4 with cord expansion. There was prominence of the body and atria of the lateral ventricles due to compensatory dilation after parenchymal volume loss (Fig. 1). There were no prior MRI studies for comparison.

**Fig. 2:**
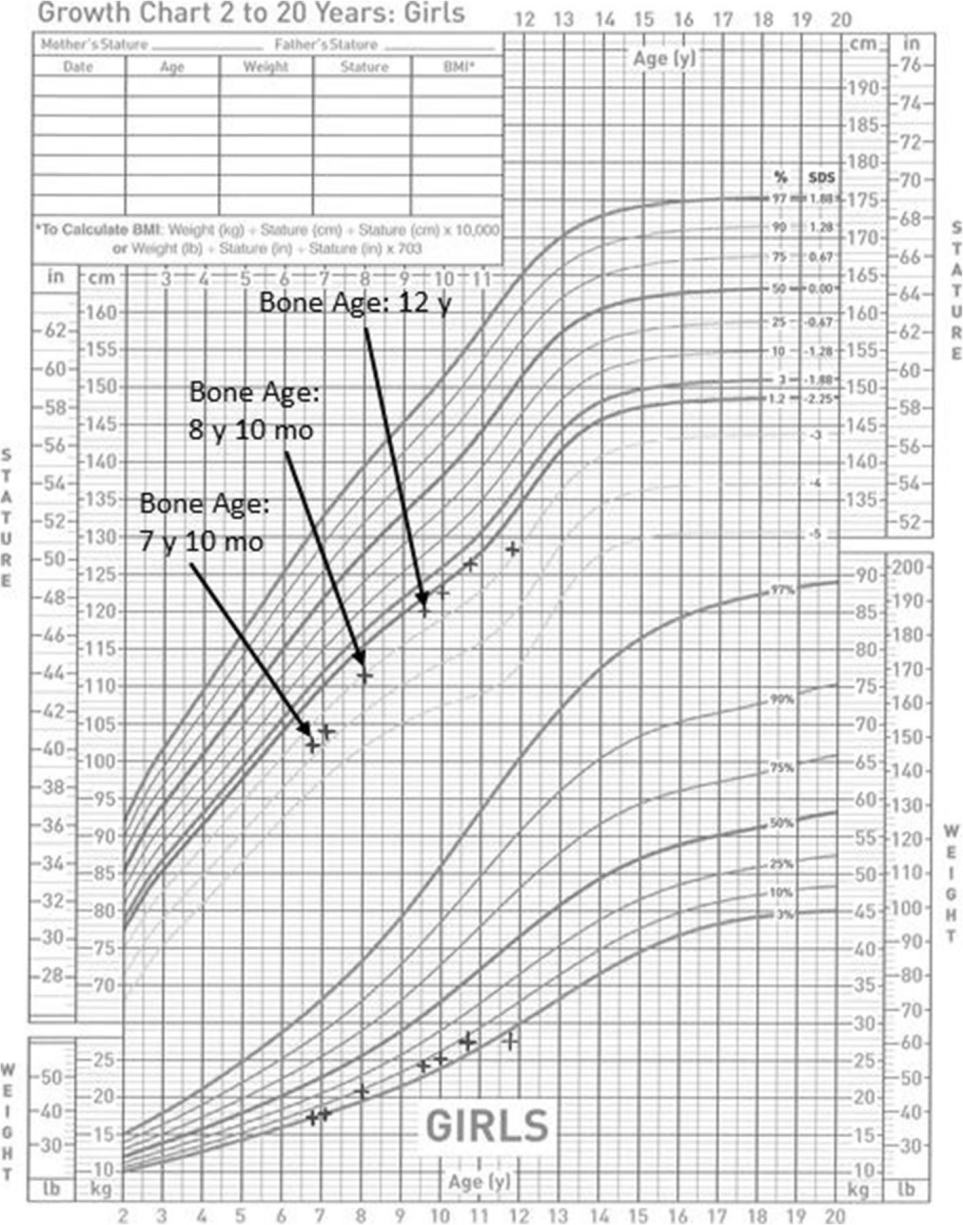
Center for Disease Control/ National Center for Health Statistics patient growth chart after presenting to pediatric endocrinology, ages 6–11 years. Stature in inches (in) and centimeters (cm), weight in pounds (lb) and kilograms (kg), age/ bone age in years (y) and months (mo), percentages (%) and standard deviation score (SDS) shown

Due to parental concern of menarche with the patient’s delayed emotional maturity, as well as the need for neurosurgical intervention, the patient was treated with a histrelin implant for 1 year, starting at 9 years 11 months. A Chiari decompression was successfully performed at 10 years 3 months, and at a follow-up visit at 10 years 9 months, the patient reported experiencing fewer headaches than prior to surgery (of note, the patient had not reported headaches on review of systems questions at prior endocrinology visits). She did not display further signs of pubertal progression. Mid-afternoon laboratory values were consistent with a suppressed LH (0.49 μIU/mL, down from 2.65 μIU/mL prior to histrelin) and estradiol (2 pg/mL, down from 7 pg/mL prior to histrelin). The histrelin implant was removed at 11 years 0 months. At 11 years 10 months, breasts were TS IV and height measured − 2.95 SD below the mean; repeat bone age x-ray was deferred. Mid-afternoon laboratory values confirmed the resumption of puberty with LH of 1.55 μIU/mL (trending up; estradiol was 7 pg/mL, consistent with the estradiol level prior to initiation of histrelin therapy); the patient remained premenarchal. Figure 2 summarizes the patient’s growth.

## Discussion and conclusions

The patient displayed an atypical presentation of CPP in CS, which is classically associated with delayed pubertal development. CPP, as defined by premature activation of the hypothalamic-pituitary-gonadal (HPG) axis before the age of 8 years in females, has a reported incidence of 1:5000-10,000. Typically, the primary concerns of CPP are short adult stature due to early epiphyseal fusion, incomplete psychosocial ability to handle early menarche and discrepancies between physical and emotional maturity [8]. These concerns are heightened in a child with CPP and CS, which is already associated with short stature and intellectual disability.

Although the vast majority of CPP in girls is idiopathic, rarely, it can be associated with monogenic conditions, tumor formation or other cranial abnormalities [8]. Recent recommendations advise against routine brain MRI in girls with CPP beginning after age 6, as literature reports cranial findings are discovered in a minority of cases of CPP, of which only 1.6% are actionable tumors requiring treatment [9].

As CPP is rare in CS, we propose children with CS presenting with borderline precocious puberty receive brain MRI. A 2003 literature review revealed 73.7% of CS patients receiving MRI displayed abnormal cranial findings at baseline, consistent with a report by Gripp, et al. of 27 out of 28 CS patients found to have brain MRI abnormalities [3, 10]. In the Gripp, et al. cohort, 96% of the patients displayed posterior fossa crowding with cerebellar tonsil herniation and 46% of the patients required neurosurgical intervention. In CS, posterior fossa crowding and cerebellar herniation is often progressive, suggesting brain growth leading to Chiari malformations and ventricular enlargement which may eventually require intervention. There are no specific guidelines for brain imaging in CS, but it has been suggested all CS patients have a baseline MRI at diagnosis and follow up as clinically indicated [10]. The presented patient emphasizes the importance of MRI in CS, even with a relatively benign presentation of CPP without other pituitary dysfunction and initial denial of headaches.

The cause of CPP is uncertain for this patient. Increased intracranial pressure, which can be associated with Chiari I malformations and syrinxes, could contribute to early activation of the HPG axis [8]. *HRAS* is involved in gonadotropin-releasing hormone receptor signaling, but given the high prevalence of delayed puberty associated with the *HRAS* p.Gly12Ser mutation, the mutation alone cannot account for CPP. Van der Kaay et al. suggests there may be unidentified genetic or environmental factors influencing the RAS-MAPK pathway in RASopathies, based on relatively frequent case reports of CPP. CS can also be associated with hypothalamic dysfunction, another factor potentially influencing the timing of pubertal development [6].

Standard assessment of pituitary hormones was challenging, as the patient was unable to attend morning appointments. Based on physical examination, LH and estradiol values were likely higher in the morning. There was no cortisol assessment but given the lack of pituitary malformation on MRI, apparently intact GH-IGF-1 axis and normal thyroid function tests, suspicion for central adrenal insufficiency was low. Her significant short stature was likely due to CS, as well as nutritional deficiencies associated with feeding difficulties during early childhood. It is also almost certain CPP led to rapid advancement in bone age between the ages of 8 years 0 months and 9 years 7 months and further impacted her ultimate adult stature.

While CPP is a rare occurrence in CS, the patient’s presentation emphasizes the importance of pubertal assessment in children with CS. It also highlights the high frequency of actionable intracranial abnormalities in CS patients and the need for brain imaging, particularly in the setting of CPP.

## Data Availability

All data analyzed during this study are included in this published article.

## Abbreviations

BMI: Body mass index
CPP: Central precocious puberty
CS: Costello syndrome
GH: Growth hormone
HPG: Hypothalamic-pituitary-gonadal
IGF-1: Insulin-like growth factor 1
LH: Luteinizing hormone
MRI: Magnetic resonance imaging
RAS-MAPK: Rat sarcoma – mitogen-activated protein kinase
SD: Standard deviation
TS: Tanner stage
